# circ‐ZNF609: A potent circRNA in human cancers

**DOI:** 10.1111/jcmm.16996

**Published:** 2021-10-26

**Authors:** Yiguan Qian, Yang Li, Rongfei Li, Tianli Yang, Ruipeng Jia, Yu‐Zheng Ge

**Affiliations:** ^1^ Department of Urology Nanjing First Hospital Nanjing Medical University Nanjing China

**Keywords:** cancers, circular RNAs, circ‐ZNF609, microRNA sponge, review

## Abstract

Circular RNAs (circRNAs) are a novel group of endogenous RNAs with a circular structure. Growing evidence indicates that circRNAs are involved in a variety of human diseases including malignancies. CircRNA ZNF609 (circ‐ZNF609), derived from the ZNF609 gene sequence, has been demonstrated to be involved in the development and progression of many diseases. circ‐ZNF609 is thought to be a viable diagnostic and prognostic biomarker for several diseases and might be a new therapeutic target, but further research is needed to accelerate clinical application. Here, we review the biogenesis and function of circRNAs and the functional roles and molecular mechanism related to circ‐ZNF609 in neoplasms and other diseases.

## 1 INTRODUCTION

Noncoding RNAs (ncRNAs) are transcribed from genes and lack protein‐coding potential. Circular RNAs (circRNAs) are a type of ncRNAs. CircRNAs were discovered in 1976^1^ and found to be produced by reverse splicing of pre‐mRNA in 1979.^2^ CircRNAs form a closed circle structure via trans‐splicing to form a covalent bond connecting the 3′ and 5′ ends.^3^ Unlike linear ncRNAs including long noncoding RNAs (lncRNAs) and microRNAs (miRNAs), circRNAs were initially thought to be accidental byproducts due to post‐transcriptional processing errors.^4^ However, with the help of high‐throughput screening methods, circRNAs are now understood to be more than transcriptional byproducts and enriched in tissues and body fluids.^5^ In addition, the biological function of circRNAs has been increasingly revealed. CircRNAs mainly act as sponges of miRNAs to regulate downstream genes transcription.^6^ CircRNAs have also been shown to be involved in various pathological processes, including the occurrence and development of cancer.^7^ Studies have indicated that circRNAs are differentially expressed in almost all cancers. Therefore, the expression pattern of circRNAs in diseases is complex, and the roles of circRNAs in different diseases have attracted increasing attention in recent years.

**FIGURE 1:**
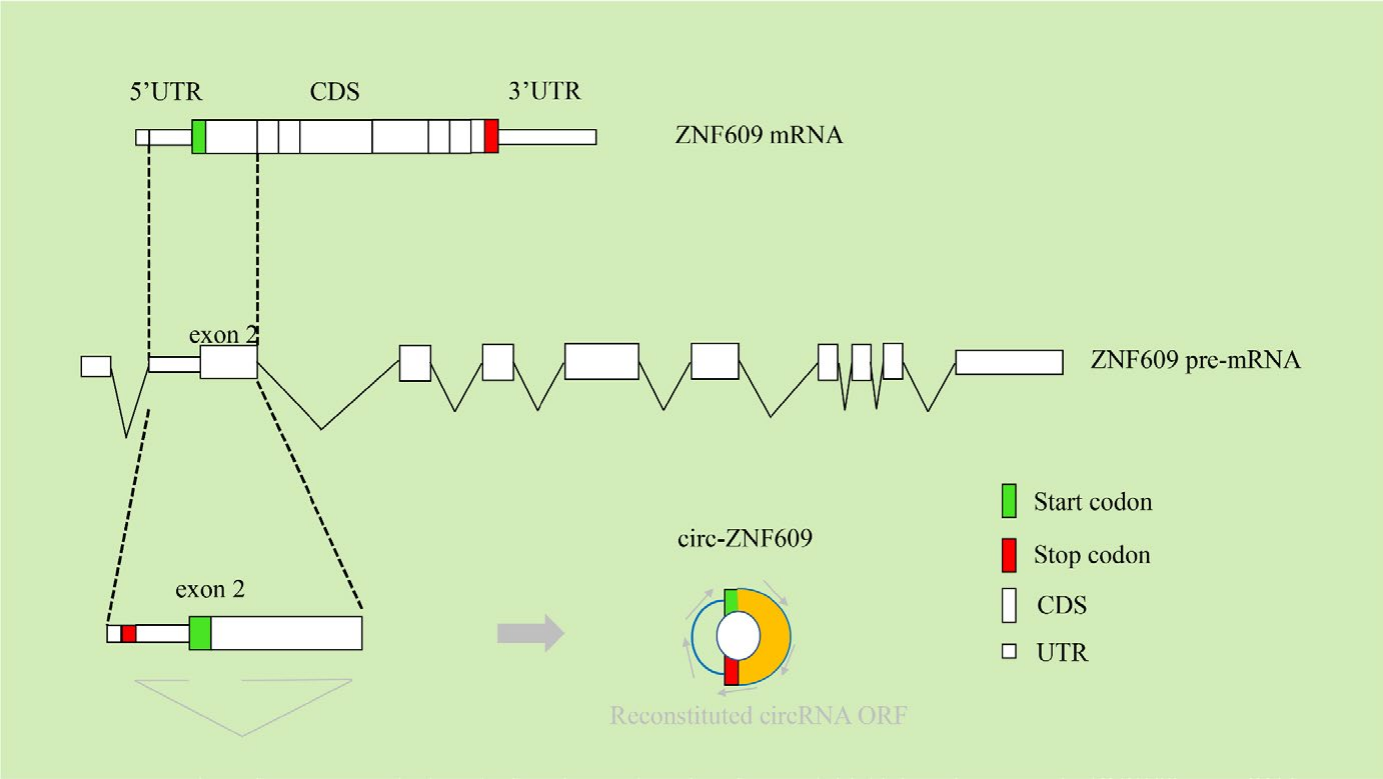
Biogenesis and conservation of circ‐ZNF609. The smaller boxes represented the 5′ and 3′ UTRs while the larger boxes indicated the Coding sequences (CDS). Start codons are shown in green while stop codons are shown in red. circ‐ZNF609 (right) originates from ZNF609 mRNA (above), ZNF609 pre‐mRNA (centre) and ZNF609 s exon (below)

CircRNA ZNF609 (circ‐ZNF609; circBase ID: hsa_circ_0000615, alias hsa_circ_000193) is located on chr15: 64791491–64792365, and the corresponding host gene is ZNF609. circ‐ZNF609 is derived from the circulation of the second exon of ZNF609 (Figure 1). circ‐ZNF609 has a 753‐nucleotide (nt) open reading frame (ORF) spanning from the putative AUG of the host gene to a STOP codon.^8^ According to the website circAtlas (version 2.0), circ‐ZNF609 is universally expressed in many species, including human, macaca, mouse, rat, pig and dog, which demonstrates that circ‐ZNF609 has high conservation and fundamental biological functions among species.^9^ circ‐ZNF609 was initially reported in the central nervous system.^10^ Recently, it has been reported that abnormal expression of circ‐ZNF609 is associated with tumours and other diseases, including renal cell carcinoma (RCC), colorectal cancer (CRC), rhabdomyosarcoma (RMS), nasopharyngeal carcinoma (NPC), gastric cancer (GC), lung cancer (LC), hepatocellular carcinoma (HCC), prostate cancer, cervical cancer (CC), glioma, Hirschsprung's disease (HSCR), vascular thyroid dysfunction, corneal neovascularization (CNV), glaucoma, neuropathic pain, myotonic dystrophy type 1 (DM1), diabetic retinopathy (DR) and lupus nephritis (LN).

In this review, we aim to describe the biological roles and molecular mechanism of circ‐ZNF609 in tumour and other diseases and further explore its diagnostic, prognostic and therapeutic value.

## 2 BIOGENESIS OF CIRCRNAS

**FIGURE 2:**
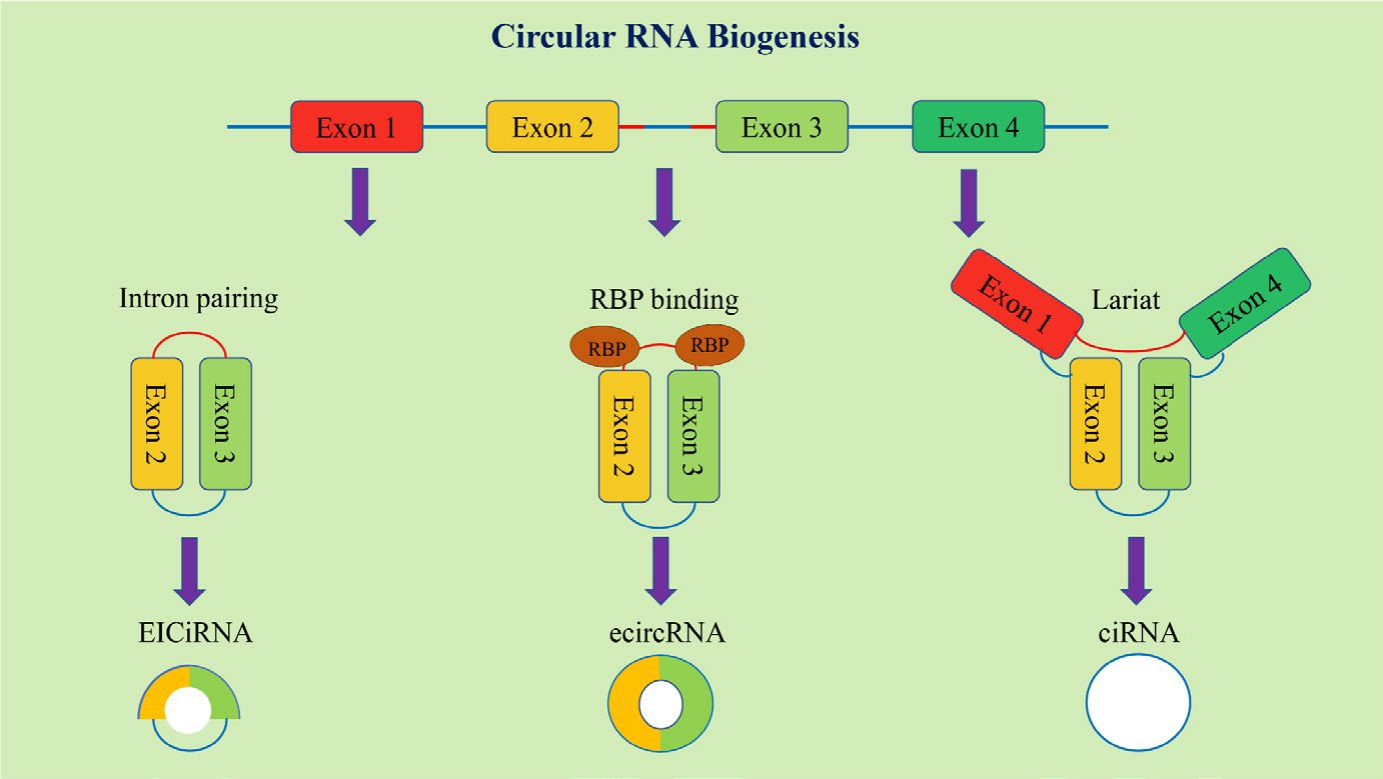
Biogenesis of circRNAs. CircRNAs are generated from lariat‐driven circulation or back‐splicing events. (Three main mechanisms: intron pairing‐driven circularization, RBPs‐mediated circularization and lariat‐driven circularization). RBPs, RNA‐binding proteins

We summarize three potential models of circRNA biogenesis according to recent studies (Figure 2). In the first model, the pre‐mRNA partially folds during transcription, prompting the 3′ splice site of the downstream intron to be attacked by the 5′ splice site of the upstream intron. Reverse splicing of the folded region produces circRNAs, and the remaining sequence forms linear RNA. Most circRNAs are produced by this mechanism.^11^ The circRNAs produced by the second model can be divided into two types: circRNAs with intron sequences retained and coexisting with exons (EIcircRNAs) and circRNAs with intron sequences removed (EcircRNAs). The circRNAs in the EcircRNA group are produced by reverse splicing via reverse complementary sequences at introns.^11^ The third model requires a common sequence in which a 7‐nt GU‐rich element is near the 5′ splice site and an 11‐nt C‐rich element is near the branchpoint site.^12^

## 3 FUNCTIONS OF CIRCRNAS

### 3.1 Biomarkers

**FIGURE 3:**
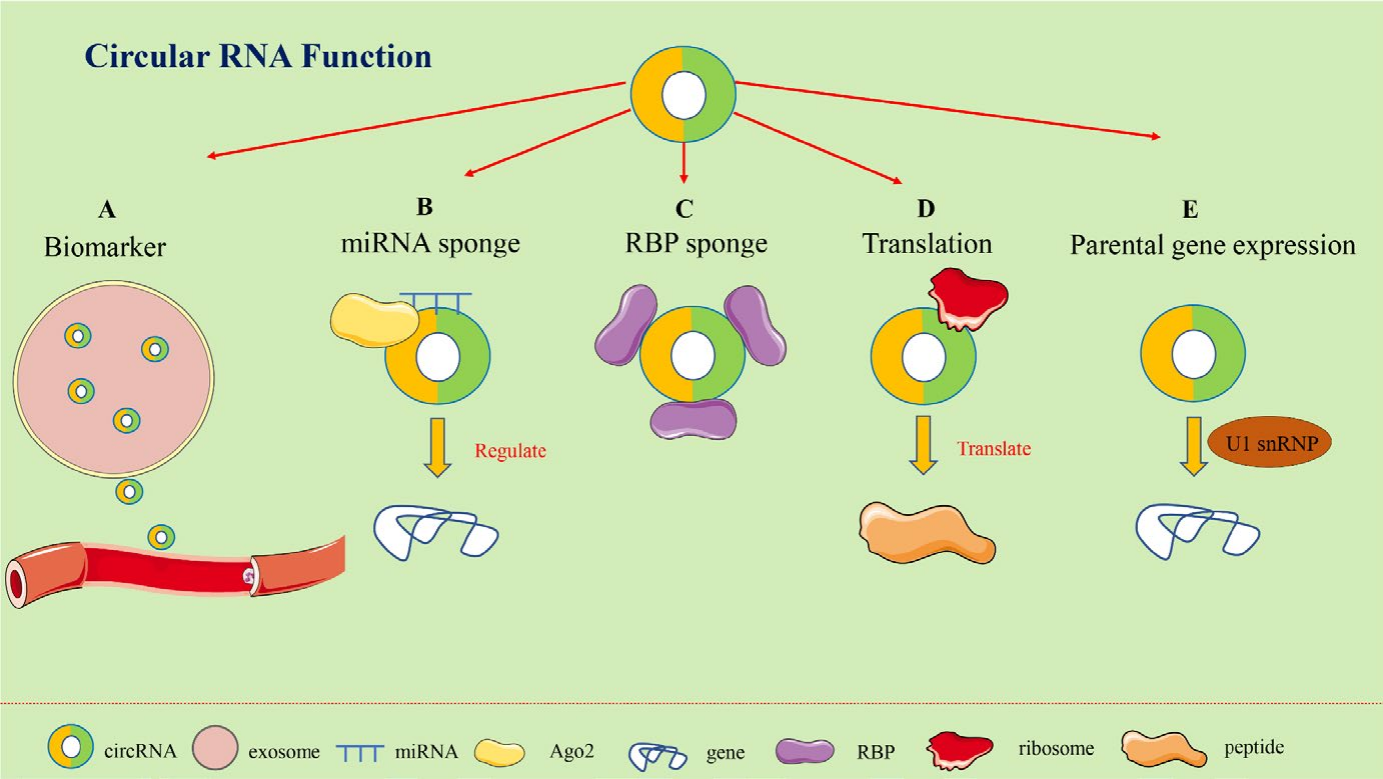
Functions of circRNAs. CircRNAs can act as biomarkers (A), miRNAs sponges (B), be combined with RBPs (C), Act as templates for protein translation (D). Abbreviations: circRNAs, circular RNAs. miRNAs, microRNAs. RBPs, RNA‐binding proteins

Currently, mounting evidence indicates that circRNAs are ideal biomarkers for the diagnosis and prognosis of many diseases, especially malignancies (Figure 3A). In pancreatic ductal adenocarcinoma (PDAC), it has been reported that the expression levels of circ‐PDE8A and circ‐IARS are correlated with tumour progression and postoperative survival time, which might be ideal biomarkers for PDAC diagnosis or progression.^13^, ^14^ In addition, exosomal circRNAs were found to be significantly upregulated in DKs‐8 cells compared with DLD‐1 and DKO‐1 cells, suggesting that exosomal circRNAs may be promising biomarkers in CRC.^15^ Lu et al.^16^ found that circ‐RanGAP1 expression was positively correlated with an advanced TNM stage and shorter survival time. Moreover, Li and colleagues^17^ found that hsa_circ_0057762 and hsa_circ_0003090 play an important role in differentiating systemic lupus erythematosus (SLE) children from healthy controls.

### 3.2 Function as miRNA sponges

One of the most widely studied function of circRNAs is serving as miRNA sponges in the cytoplasm (Figure 3B).^18^ CircRNAs have been proven to be involved in various diseases as competitive endogenous RNAs (ceRNAs).^19^, ^20^ In DR, circular RNA ZNF532 promoted the viability, proliferation, and differentiation of pericytes and elevated the recruitment of pericytes towards endothelial cells by acting as a miR‐29a‐3p sponge and inducing increased expression of NG2, LOXL2 and CDK2.^21^ In osteoarthritis, circ‐SERPINE2 could act as a sponge of miR‐1271‐5p and functioned in human chondrocytes (HCs) through targeting miR‐1271‐5p and ERG.^22^ Yu et al.^23^ found that circ‐BIRC6 was enriched in the AGO2 complex and directly interacts with miR‐34a‐3p and miR‐145‐3p to attenuate the downregulation of these target genes and suppressed human embryonic stem cells differentiation. Besides, Zheng et al.^24^ generated ribosomal‐depleted RNA sequencing data from normal and cancer tissues, and detected at least 27,000 circRNA candidates. They demonstrated that circ‐HIPK3 was able to sponge 9 miRNAs with 18 potential binding sites, which might have a regulatory role in cancers.

### 3.3 Interaction with proteins

CircRNAs can form circRNA‐protein complexes (circRNPs) by interacting with different proteins, thereby regulating the mode of action of proteins or the expression of target genes (Figure 3C). For example, circ‐MBL can interact with mannose‐binding lectin (MBL) and modulate MBL and circ‐MBL via negative feedback.^25^ circ‐PABPN1 can bind to HuR, which inhibits the binding of HuR to PABPN1 mRNA, thus reducing the translation of PABPN1.^26^ Moreover, circular RNA DNA methyltransferase 1 (circ‐DNMT1) binds to p53 and AU binding factor 1 (AUF1), thereby accelerating breast cancer progression.^27^

### 3.4 CircRNAs as templates for protein translation

In the past, circRNAs were thought as lack of protein translation potential. However, many recent reports have shown that circRNAs with ORFs and an internal ribosome entry site (IRES) can be efficiently translated into peptides or proteins (Figure 3D). The IRES, which initiates protein translation independently of the 5′‐cap structure, is a specific nucleotide sequence in endogenous circRNAs.^28^ For example, circ‐FAM188B encodes a novel protein called circ‐FAM188B‐103aa, which was suggested to promote proliferation but inhibit differentiation of chicken skeletal muscle satellite cells.^29^ Gprc5a is an essential protein in bladder cancer stem cells. However, in the absence of a protein encoded by circ‐Gprac5a, Gprc5a did not function, which indicated that the circ‐Gprc5a‐peptide‐Gprc5a pathway was closely associated with bladder cancer progression.^30^ In addition, Yang et al.^31^ demonstrated that N^6^‐methyladenosine can promote circRNAs to effectively initiate protein translation in the response of cells to environmental stress.

### 3.5 CircRNAs affect parental gene expression

Emerging evidence has demonstrated that some circRNAs retained in the cell nucleus could affect their parental gene expression (Figure 3E). Li et al.^32^ found that circ‐EIF3J and circ‐PAIP2 could interact with RNA Pol II and promote their parental gene transcription in interaction with U1 snRNP in the nucleus of cells. Another study indicated that circ‐ANKRD52 could specifically combine with the elongation RNA Pol II complex and directly promote ANKRD52 transcription.^12^ Moreover, overexpression of circ‐Yap could suppress the interaction of PABP with eIF4G and consequently inhibit the translation initiation of Yap mRNA.^33^ circ‐PABPN1 might repress HuR binding to PABPN1 mRNA and hinder the translation of PABPN1.^26^ These studies verified that circRNAs might affect their parental gene expression at both the transcriptional and translational levels.

### 3.6 circ‐ZNF609 in cancers and other diseases

Recently, emerging evidence has demonstrated that circ‐ZNF609 is differentially expressed in distinct cancers, such as RCC, CRC, NPC, GC, LC, HCC, prostate cancer, CC, glioma and other diseases such as RMS, HSCR, vascular dysfunction, glaucoma, CNV, neuropathic pain, DM1, DR and LN. The functional role and potential molecular mechanisms involved are listed in Tables 1 and 2.

**TABLE 1:** Functional characterization of circ‐ZNF609 in various malignancies

Tumour types	Expression	Role	Function role	miRNAs	Related genes	References
Renal cell carcinoma	Upregulation	Oncogene	Proliferation and invasion	miR‐138‐5p	FOXP4	36
Colorectal cancer	Upregulation	Oncogene	Proliferation, migration, invasion and apoptosis	miR‐150‐5p	Gli1	39
Rhabdomyosarcoma	Upregulation	Not investigated				42
Nasopharyngeal carcinoma	Upregulation	Oncogene	Proliferation and metastasis	miR‐150‐5p	Sp1	46
Nasopharyngeal carcinoma	Upregulation	Oncogene	Proliferation, migration, invasion and glycolysis	miR‐338‐3p	HRAS	47
Nasopharyngeal carcinoma	Upregulation	Oncogene	Proliferation, migration and angiogenesis	miR‐145‐5p	STMN1	48
Gastric cancer	Upregulation	Oncogene	Proliferation and invasion	miR‐145‐5p	/	52
Lung Adenocarcinoma	Upregulation	Oncogene	Proliferation	miR‐1224‐3p	EVT1	57
Non‐small cell lung cancer	Upregulation	Oncogene	Viability, migration, invasion and apoptosis	miR‐623	FOXM1	58
Lung cancer	Upregulation	Oncogene	Proliferation and migration	miR‐142‐3p	GNB2	59
Hepatocellular carcinoma	Upregulation	Oncogene	Migration, invasion and cell apoptosis	miR‐342‐3p	RAP2C	63
Prostate cancer	Upregulation	Oncogene	Viability, metastasis, radioresistance and apoptosis	miR‐501‐3p	HK2	65
Cervical cancer	Upregulation	Oncogene	Proliferation, migration and invasion	miR‐197‐3p	E2F6	70
Glioma	Upregulation	Oncogene	Proliferation, migration and invasion	miR‐1224‐3p	PLK1	72

**TABLE 2:** Functional characterization of circ‐ZNF609 in non‐cancer diseases

Diseases types	Expression	Role	Function role	miRNAs	Related genes	References
Hirschsprung	Downregulation	Disease suppressor	Proliferation, differentiation and apoptosis	miR‐150‐5p	AKT3	75
Vascular dysfunction	Upregulation	Disease promoter	Migration, tube formation and apoptosis	miR‐615‐5p	MEF2A	79
Corneal neovascularization	Upregulation	/	Cell growth, migration and tube formation	miR‐184	AKT/b‐catenin/VEGF	82
Glaucoma	Upregulation	Disease promoter	Not investigated	miR‐615‐5p	METRN	86
Neuropathic pain	Downregulation	Disease suppressor	Not investigated	miR‐22‐3p	ENO1	89

### 3.7 circ‐ZNF609 in cancers

**FIGURE 4:**
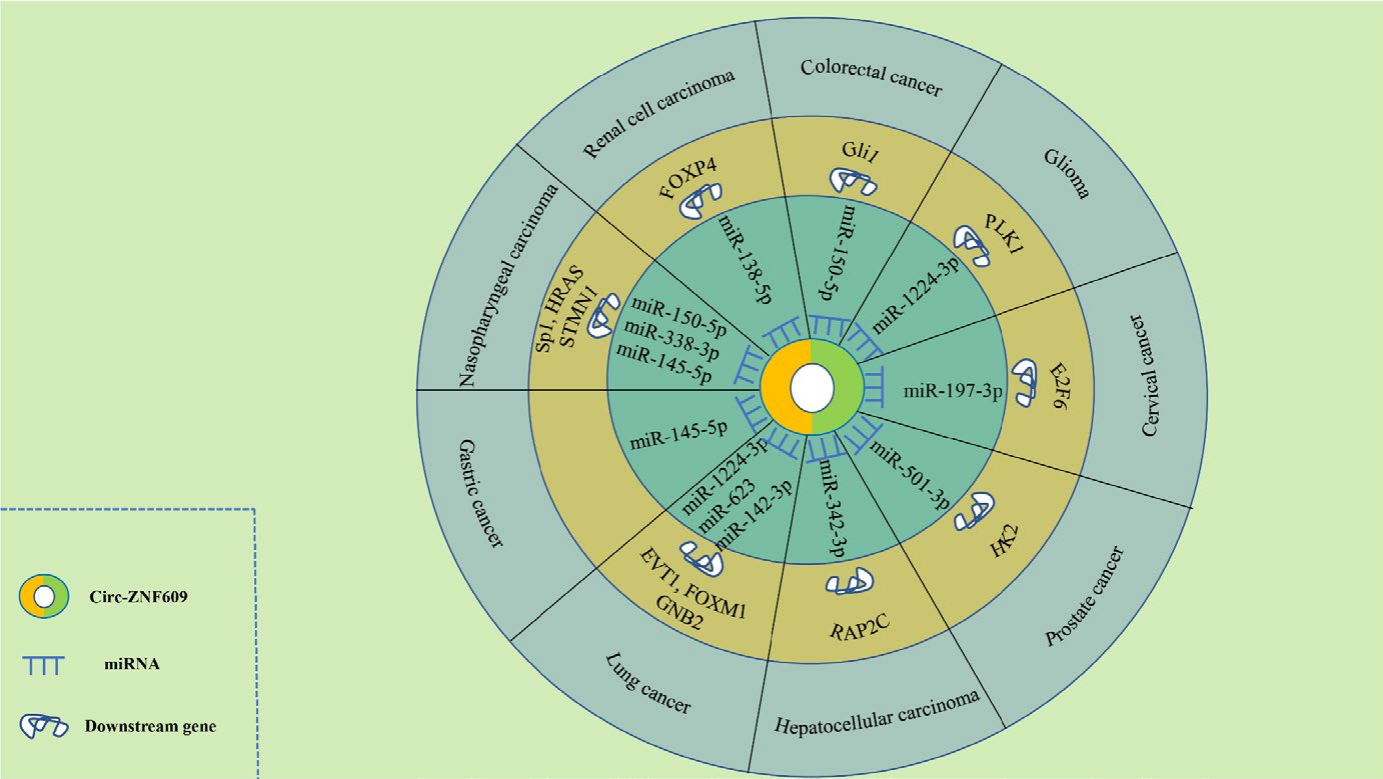
circ‐ZNF609 mediates mechanisms involved in cancer progression. (RCC) circ‐ZNF609 could upregulate the expression of FOXP4 by sponging miR‐138‐5p. (Nasopharyngeal carcinoma) circ‐ZNF609 may act as a ceRNA to sponge miR‐150‐5p, miR‐338‐3p or miR‐145‐5p to promote the expression of SP1, HRAS or STMN1. (Gastric cancer) circ‐ZNF609 could bind to miR‐145‐5p. (Lung cancer) circ‐ZNF609 can promote ETV1, FOXM1 or GNB2 signal pathway through sponging miR‐1224‐3p, miR‐623 or miR‐142‐3p. (Hepatocellular carcinoma) circ‐ZNF609 could promote RAP2C expression via serving as sponge of miR‐342‐3p. (Prostate cancer) circ‐ZNF609 could sponge miR‐501‐3p to promote the HK2 expression. (Cervical cancer) circ‐ZNF609 may promote the expression of E2F6 via binding to miR‐197‐3p. (Glioma) circ‐ZNF609 upregulated the PLK1 expression by sponging miR‐1224‐3p. (Colorectal cancer) circ‐ZNF609 may promote Gli1 signal pathway through sponging miR‐150‐5p. FOXP4, forkhead box P4. HRAS, HRas proto‐oncogene, GTPase. STMN1, stathmin 1. ETV1, ets variant gene 1. FOXM1, forkhead box M1. GNB2, G protein subunit beta 2. HK2, hexokinase 2. E2F6, E2F transcription factor 6. PLK1, Polo‐like Kinase 1

Mounting evidence has demonstrated that circ‐ZNF609 could exert vital functions in a variety of human malignancies. circ‐ZNF609 can act as ceRNA to regulate several target genes via sponging different miRNAs, so as to participate in the initiation and development of cancers (Figure 4).

#### 3.7.1 Renal cell carcinoma (RCC)

RCC is the third most common malignant tumour of the genitourinary system.^34^ Although numerous studies have focused on identifying molecular biomarkers of RCC, few of these markers have been proven useful in determining disease prognosis and applied in clinical practice.^35^ Therefore, it is urgent to find new biomarkers and therapeutic targets for RCC.

Xiong et al.^36^ found that circ‐ZNF609 expression was significantly increased in RCC tissues and cell lines. In vitro experiments showed that silencing circ‐ZNF609 had a negative effect on cell proliferation and invasion. Further molecular assays demonstrated that the circ‐ZNF609/miR‐138‐5p/FOXP4 regulatory axis was involved. In summary, the preliminary study concluded that circ‐ZNF609 exerts an important role in the pathogenesis of RCC by regulating FOXP4 expression through sponging of miR‐138‐5p.

#### 3.7.2 Colorectal cancer (CRC)

CRC is a common malignant tumour worldwide that has a high death rate and molecular heterogeneity. The incidence and mortality of CRC rank at near the top, and the incidence of CRC in younger patients appears to be increasing.^37^ Although multiple treatments for CRC are available, the prognosis of CRC patients remains poor.^38^ Therefore, exploration of potential mechanism and early diagnostic biomarker are key and urgent.

Wu et al.^39^ found that circ‐ZNF609 and Gli1 were highly expressed in CRC tissues. Knockdown of circ‐ZNF609 had a negative impact on the migration of HCT116 cells. Further study showed that circ‐ZNF609 could regulate Gli1 expression through miR‐150‐5p, thus affecting cancer metastasis. circ‐ZNF609 might be a novel target for subsequent CRC screening and treatment, while the downstream target genes for this pathway remains unexplored.

#### 3.7.3 Rhabdomyosarcoma (RMS)

RMS is one of the most common soft tissue sarcomas in children, accounting for 4.5% of all paediatric malignant tumours.^40^ Although a combination of surgery, radiotherapy and chemotherapy is widely recommended in RMS and has consequently improved the 5‐year survival rate of most children, the prognosis of high‐risk cases is still poor.^41^ Therefore, exploring the genes participating in the pathogenesis of RMS and clarifying their functional roles in RMS will provide new approaches for the diagnosis and treatment of RMS.

Francesca Rossi et al. previously reported that circ‐ZNF609 exhibited fundamental roles in maintaining the growth of human primary myoblast cells. Knocking down circ‐ZNF609, rather than its linear counterpart, significantly reduced the proliferation rate of myoblasts cultured.^8^ circ‐ZNF609 was specifically upregulated in two major RMS subtypes, embryonal RMS (ERMS) and alveolar RMS (ARMS). Subsequently, the investigators knocked out circ‐ZNF609 in ERMS‐derived cell lines and found specific blockade during the G1‐S transition, which was accompanied by a significant decrease in the p‐Akt protein level and changes in the pRb/Rb ratio. Interestingly, circ‐ZNF609 inhibition had no obvious effect on the cell cycle in cell lines derived from ARMS. The researchers further demonstrated that circ‐ZNF609 might participate in disease progression via inhibiting p‐Akt proteasomal‐dependent degradation. However, the mechanism by which circ‐ZNF609 regulates Rb, pRb and p‐Akt levels remains far from understood, and further studies are needed.^42^

#### 3.7.4 Nasopharyngeal carcinoma (NPC)

NPC is one of the most frequently diagnosed head and neck cancer types. Radiotherapy is the first‐line treatment for NPC.^43^ In recent years, with the progress in radiotherapy technology and the improvement in comprehensive treatments combining radiotherapy and chemotherapy, the overall survival (OS) rate and local control rate for NPC patients have improved to some extent, but the distant metastasis rate remains high.^44^, ^45^ Therefore, finding specific markers that can predict the occurrence and development of NPC is urgent.

To date, three studies have found that circ‐ZNF609 was highly expressed in NPC tissues, but their mechanisms of action are different. Zhu et al.^46^ found that high expression levels of circ‐ZNF609 and Sp1 could promote the proliferation, migration and invasion of NPC cells, while high levels of miR‐150‐5p exerted the opposite effects. The researchers further found that circ‐ZNF609 could elevate the expression of the oncogene Sp1 by binding to miR‐150‐5p, thus accelerating the progression of NPC. Liu et al.^47^ reported high circ‐ZNF609 expression contributed to a poor prognosis and circ‐ZNF609 regulated HRAS through miR‐338‐3p to promote the progression of NPC. Wang et al.^48^ found that silencing circ‐ZNF609 inhibited proliferation, migration and angiogenesis of NPC cells, while miR‐145‐5p inhibition reversed these effects. Furthermore, STMN1, the downstream target of miR‐145‐5p and circ‐ZNF609, promotes angiogenesis in NPC. Therefore, circ‐ZNF609 might have the potential to act as a diagnostic marker and therapeutic target in NPC.

#### 3.7.5 Gastric cancer (GC)

GC is a malignant neoplasm with high heterogeneity. Its pathogenesis is mainly related to genetic variations, Helicobacter pylori and Epstein‐Barr virus infection, and epigenetic changes.^49^ At present, surgical resection with chemotherapy is one of the conventional treatment approaches for GC patients. A series of chemotherapeutic and molecular‐targeted drugs, including 5‐fluorouracil or oxaliplatin followed by Herceptin, are routinely recommended.^50^ However, due to widespread toxicity/side effects, the prognosis remains far from optimistic.^51^ Therefore, it is necessary to identify novel molecular therapeutic targets for GC and explore their correlation with the prognosis of GC to further optimize precision therapy for GC patients.

Liu et al.^52^ demonstrated that circ‐ZNF609 was expressed higher in GC tissues compared to the normal tissues. circ‐ZNF609 facilitated the proliferation and invasion of GC cells, while miR‐145‐5p exerted the opposite effect. In addition, knockdown of circ‐ZNF609 in BGC823 and MGC803 cells suppressed cell proliferation and invasion. Further studies suggested that circ‐ZNF609 can competitively bind to miR‐145‐5p without detailed binding site, thus preventing the degradation of downstream target genes induced by miR‐145‐5p and indirectly upregulating the expression of target genes.

#### 3.7.6 Lung cancer (LC)

In 2020, with 2.2 million new LC cases and 1.8 million deaths reported worldwide, LC is the second most commonly diagnosed cancer and the leading cause of cancer death.^53^ LC is pathologically classified into small cell lung cancer (SCLC) and non‐small cell lung cancer (NSCLC),^54^ while NSCLC can be further divided into three subtypes: large cell lung carcinoma, lung squamous cell carcinoma and lung adenocarcinoma (LUAD).^55^ Recently, despite significant advances in the diagnosis and treatment of LC have been achieved, there are few valuable molecular biomarkers, and the prognosis of LC patients remains dismal, with a 5‐year OS rate of only 16.6%.^56^ Therefore, it is of great value to explore the mechanism and novel targets for different LC types.

Zuo et al.^57^ reported that circ‐ZNF609 was significantly upregulated in LUAD tissues compared with normal tissues. Inhibition of circ‐ZNF609 significantly repressed the proliferation of LUAD cells. Mechanistically, circ‐ZNF609 restored expression of the downstream target gene EVT1 by binding to miR‐1224‐3p, and this effect could be reversed by transfection with miR‐1224‐3p mimics. With more strong theoretical support to the sponge hypothesis, circ‐ZNF609/miR‐1224‐3p/EVT1 axis might be a potential therapeutic target in LUAD. Wang et al.^58^ also found that circ‐ZNF609 abundance was enhanced in NSCLC tissues and high expression of circ‐ZNF609 were associated with poor prognosis. The mechanism studies showed that knockdown of circ‐ZNF609 inhibited the development of NSCLC through miR‐623/FOXM1 axis. Liu et al.^59^ also reported that circ‐ZNF609 was markedly upregulated in LC tissues compared with adjacent normal lung tissues and circ‐ZNF609 could facilitate the proliferation and migration in LC cells. Differed from the previous studies, they further found that FUS RNA‐binding proteins bind to pre‐ZNF609 RNA, inducing circ‐ZNF609 formation and increasing circ‐ZNF609 expression in LC cells. Mechanistically, FUS‐induced circ‐ZNF609 exerts promotional effects on LC cells proliferation and migration through modulation of the miR‐142‐3p/GNB2 axis. According to the current studies, circ‐ZNF609 plays an oncogenic role in different types of LC. However, the clinical sample size of these studies is insufficient, we are looking forward to the follow‐up verification with large samples.

#### 3.7.7 Hepatocellular carcinoma (HCC)

HCC is a malignant tumour that seriously threatens human health. Approximately 750,000 new cases are diagnosed every year, and the 5‐year OS rate is 5%~9%.^60^ The effect of surgery and chemoradiotherapy is not satisfactory. With the development of molecular biology techniques and the application of targeted therapy, it is imperative to study the molecular mechanism underlying the occurrence and development of HCC and find the corresponding effective therapeutic targets.^61^, ^62^

Liao and colleagues^63^ first found that circ‐ZNF609 was overexpressed in HCC tissues and positively correlated with poor prognosis. In vitro experiments showed that knockout of circ‐ZNF609 effectively suppressed the activity, migration and invasion of cancer cells. Simultaneously, in vivo experiments showed that knockout of circ‐ZNF609 inhibited tumour growth and metastasis in mice. It was further found that the effect of circ‐ZNF609 on tumour cells was mainly realized through the circ‐ZNF609/miR‐342‐3p/RAP2C regulatory axis.

#### 3.7.8 Prostate cancer

Prostate cancer is one of the most common malignant tumours of the urinary and male reproductive systems in middle‐aged and elderly men. Prostate cancer is usually asymptomatic in its early stage, and most patients are diagnosed in the advanced stages. Endocrine therapy is the preferred treatment for advanced prostate cancer. However, for castration‐resistant prostate cancer, endocrine therapy is less effective.^64^ At present, the molecular mechanism of prostate cancer remains elusive. Therefore, in‐depth study of the key genes related to prostate cancer is of great significance for the early diagnosis and targeted therapy.

Du and colleagues^65^ confirmed that circ‐ZNF609 is abnormally upregulated in prostate cancer tissues and cell lines. Silencing of circ‐ZNF609 inhibited cell survival, metastasis, radiation tolerance and apoptosis by inhibiting glycolysis. An important finding in prostate cancer is that silencing circ‐ZNF609 increases the radiosensitivity of prostate cancer cells in vivo. In terms of the mechanism, miR‐501‐3p was a direct target of circ‐ZNF609, and HK2 was an indirect target of circ‐ZNF609. HK2 was regulated by the circ‐ZNF609/miR‐501‐3p axis in prostate cancer cells, which affected the survival, metastasis, radiation resistance and apoptosis of prostate cancer cells. Study of circ‐ZNF609 in prostate cancer will provide a novel potential target for the treatment of prostate cancer, although further exploration is needed.

#### 3.7.9 Cervical cancer (CC)

CC is the most common gynaecological malignant tumour and the fourth leading cause of cancer death in women.^66^ It is estimated that there were 604,000 new cases of CC and 342,000 deaths worldwide in 2020.^53^ In present, studies have confirmed that high‐risk human papillomavirus (HPV) infection is the main cause of CC, about 95% of CC is related to HPV infection, leading to precancerous lesions and invasion of CC.^67^ Recently, NF‐YA and EphA2 were identified as key regulators in the pathogenesis of CC, without detailed exploration in the clinical setting.^68^, ^69^ Therefore, it is of great clinical significance to deeply explore the pathogenesis of CC and find more potential molecular markers of CC.

Gu et al.^70^ found that circ‐ZNF609 was significantly upregulated in CC. Further experiments demonstrated that circ‐ZNF609 promoted malignant phenotypes in CC cells, and miR‐197‐3p could reverse the effects of circ‐ZNF609. circ‐ZNF609 functioned as a miRNA sponge to positively regulate the expression of E2F transcription factor 6 (E2F6) through sponging miR‐197‐3p and subsequently promoted malignant phenotypes of CC cells. However, this paper relied only on the use of luciferase assays in the presence of mimics to validate the direct circZNF609‐miRNA interaction without pull‐down assay.

#### 3.7.10 Glioma

Glioma is the most common primary intracranial tumour. Glioma can be classified into low‐grade and high‐grade subtypes. High‐grade glioma is characterized by rapid invasive growth, vascular dysplasia and destruction of brain tissue, which make it difficult to treat and have a poor prognosis.^71^ Currently, surgical resection with radiotherapy and chemotherapy is the standard of treatment for glioma patients, but the OS is not satisfactory. Therefore, it remains an urgent priority to identify the potential pathogenesis and treatment of glioma.

Du et al.^72^ observed that circ‐ZNF609 was upregulated in glioma. Elevated circ‐ZNF609 expression led to increased migration and invasion of glioma cells both in vitro and in vivo whereas silencing circ‐ZNF609 decreased migration and invasion of glioma cells. Mechanistically, circ‐ZNF609 could directly bind miR‐1224‐3p and further promote the expression of target gene PLK1. However, this paper relied only on the use of luciferase assays in the presence of mimics to explore the interacting miRNAs of circ‐ZNF609, and more experiments such as pull‐down assays can be added in future to increase the credibility.

### 3.8 Circ‐ZNF609 in human non‐malignant diseases

**FIGURE 5:**
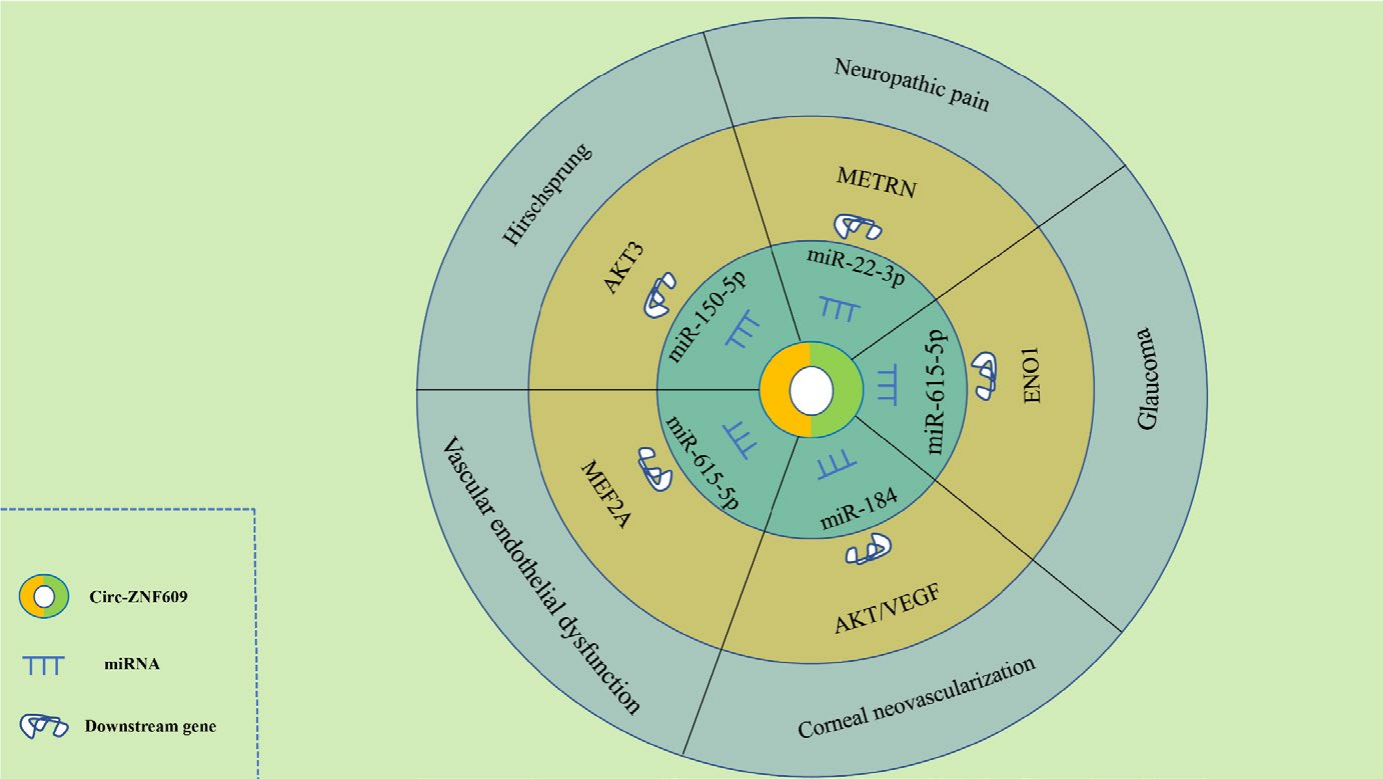
circ‐ZNF609 mediates mechanisms involved in human non‐malignant diseases progression. (Hirschsprung) circ‐ZNF609 could upregulate the expression of AKT3 by sponging miR‐150‐5p. (Vascular endothelial dysfunction and Glaucoma) circ‐ZNF609 upregulated the MEF2A and METRN expression by sponging miR‐615‐5p. (Corneal neovascularization) circ‐ZNF609 may promote the expression of AKT and VEGF via binding to miR‐184. (Neuropathic pain) circ‐ZNF609 may promote ENO1 signal pathway through sponging miR‐22‐3p. AKT3, AKT serine/threonine kinase 3. MEF2A, myocyte enhancer factor 2A. METRN, meteorin. ENO1, enolase 1

Moreover, circ‐ZNF609 exerts an essential role in various non‐tumour diseases under different molecular mechanisms (Figure 5).

#### 3.8.1 Hirschsprung's disease (HSCR)

HSCR is caused by the abnormal development of intestinal neurons. The incidence of HSCR in males is four times higher than that in females, and HSCR is a common digestive tract malformation in paediatrics. At present, the absence of ganglion cells is generally believed to be related to a halt in the development of the intestinal nervous system, which is caused by disordered migration of neural crest cells, resulting in loss or abnormal development of ganglion cells from the intermuscular plexus of the intestinal wall and the submucosal nerve.^73^ To date, fourteen HSCR‐related genes have been identified, but most cases still cannot be explained at the mechanistic level. Thus, the molecular mechanism of HSCR remains to be further explored.^74^

Peng et al.^75^ found that the expression of circ‐ZNF609 in the HSCR stenotic segment was downregulated compared with intestinal expression in normal patients. Inhibition of circ‐ZNF609 suppressed cell migration and proliferation in HSCR cell lines. Subsequent experimental evidence supported the hypothesis that circ‐ZNF609 might act as a sponge for miR‐150‐5p to regulate the expression of the downstream target gene AKT3. circ‐ZNF609 has the potential to be a new approach for the diagnosis and treatment of HSCR, and further studies such as fluorescence in situ hybridization (FISH) assay to explore the localization of circ‐ZNF609 and miRNAs are needed.

#### 3.8.2 Vascular endothelial dysfunction

Vascular endothelial dysfunction plays a key role in the progression of various malignant, inflammatory, ischaemic, infectious and immunological diseases.^76^, ^77^ These diseases are associated with vascular endothelial barrier destruction, vascular leakage, inflammation response and angiogenesis.^78^ Therefore, studying the mechanism of vascular endothelial dysfunction is of great value in the prevention and treatment of vascular complications.

Liu et al.^79^ found that circ‐ZNF609 was significantly upregulated in high‐glucose and low‐oxygen environment in vivo and in vitro. Silencing of circ‐ZNF609 reduced retinal vascular loss and pathological angiogenesis in vivo. Mechanistically, circ‐ZNF609 might act as an endogenous miR‐615‐5p sponge to increase the expression of the downstream target gene MEF2A. Importantly, the authors detected a high level of circ‐ZNF609 expression in clinical samples from patients with diabetes, hypertension and coronary artery disease, suggesting that targeted regulation of circ‐ZNF609 expression is a potential approach for the treatment of vascular dysfunction.

#### 3.8.3 Corneal neovascularization (CNV)

Under normal circumstances, the cornea is in an avascular state, with peripheral blood vessels terminating at the limbus of the cornea and forming a vascular network. The avascular state of the cornea is the key to maintaining corneal transparency and ensuring good vision. In the pathological state, new capillaries invade the cornea from the limbus, leading to the formation of CNV. According to statistics, 4.14% of ophthalmic patients in the United States exhibit CNV.^80^ The formation and eventual blindness related to CNV caused by various ophthalmic diseases have become important problems in the ophthalmic field.^81^ Therefore, explorations of the mechanism underlying CNV and identification of therapeutic targets are urgently needed.

Wu et al.^82^ found that circ‐ZNF609 and miR‐184 intervention might be promising therapeutic measures for pathologic CNV. They found that circ‐ZNF609 is highly expressed in CNV tissues while miR‐184 is lowly expressed. High expression of miR‐184 can maintain the proliferation and migration of human corneal epithelial keratinocytes cells and inhibit the formation of blood vessels in vivo, while circ‐ZNF609 exerted the opposite effect. In terms of the mechanism, circ‐ZNF609 could interacted with miR‐184 to regulate the downstream AKT and VEGF signalling pathways.

#### 3.8.4 Glaucoma

Glaucoma is the most irreversible blindness‐inducing disease in the world and seriously affects the visual health of humans. It is estimated that the number of cases of glaucoma will increase to 112 million in 2040.^83^ This is a disease characterized by progressive loss of retinal ganglion cells (RGCs), optic nerve atrophy and visual field defects. The mechanisms of elevated intraocular pressure (IOP) and nerve injury are not yet fully understood. Lowering IOP is still the main treatment strategy for glaucoma. However, progressive vision loss is common, despite effective medications and surgery to reduce IOP.^84^, ^85^ Therefore, it is urgent to study the pathogenesis of glaucoma and develop new treatment methods.

During exploring neuroprotective treatment for glaucoma, Wang et al.^86^ found that circ‐ZNF609 was highly expressed in retinal neurodegenerative lesions and played a vital role. Silencing of circ‐ZNF609 inhibited retinal RGC proliferation and glial cell activation and promoted RGC survival in glaucoma. The mechanism of action of circ‐ZNF609 was mediated through the circ‐ZNF609/miR‐615‐5p/METRN axis, and circ‐ZNF609 might bind miR‐615‐5p to manipulate the expression of METRN.

#### 3.8.5 Neuropathic pain

Neuropathic pain is initiated and maintained by mediators released by neurons and glial cells that cause neuronal sensitization in the peripheral and central nervous systems.^87^ Currently, the main treatment for neuropathic pain is pain relief with drugs. Approximately 50% of patients fail to achieve the expected effects, and many adverse events occur.^88^ Therefore, it is of urgency to understand the pathogenesis of neuropathic pain and identify alternative therapeutic targets.

Li et al.^89^ found that miR‐22‐3p and its downstream target ENO1 contribute to neuropathic pain by regulating inflammatory factors, such as TNF‐α, IL‐1 and IL‐6. Moreover, downregulation of miR‐22‐3p can promote the progression of neuropathic pain. After further investigation, it was found that the upstream regulator of miR‐22‐3p was circ‐ZNF609, and it was further found that circ‐ZNF609 could promote the progression of neuropathic pain. The discovery of the circ‐ZNF609/miR‐22‐3p/ENO1 axis provides a new perspective for the study of neuropathic pain. However, the study was limited in a rat model without further exploration in human beings.

#### 3.8.6 Other non‐cancerous diseases

With the development of high‐throughput screening technology, high expression of circ‐ZNF609 has been found in some other benign diseases. Christine Voellenkle et al.^90^ found that four transcripts (CDYL, HIPK3, RTN4_03 and circ‐ZNF609) were increased in biopsies of skeletal muscle from DM1 patients. Moreover, the expression of circ‐ZNF609 was also increased in differentiated myogenic cell lines from DM1 patients. Zhang et al.^91^ used circRNA microarray to detect the circRNA expression profiles in diabetic and nondiabetic human retinas, found that circ‐ZNF609 was upregulated in DR. Luan et al.^92^ reported the high expression of circ‐ZNF609 in LN tissues compared to normal kidney tissues, which was significantly correlated with clinical parameters of LN disease activity. However, the above‐mentioned studies only identified circ‐ZNF609 expression patterns in these diseases, and the biological functions and mechanisms remain to be studied.

## 4 CONCLUSIONS AND FUTURE PERSPECTIVES

There are still some unsolved questions about circRNA biology. At present, we know little about the reasons why circRNAs are dysregulated in diseases. It has been speculated that the abnormal expression of circRNAs may be explained in part by genetic and/or epigenetic changes, such as mutations in spliceosomal genes, but evidence is lacking.^93^ The second issue is the sensitivity of circRNAs to post‐transcriptional modifications. If abnormal modifications occur during disease, the activity of circRNAs may be inhibited or enhanced.^93^

According to recent studies on circ‐ZNF609, abnormal expression of circ‐ZNF609 is associated with the progression of various diseases, especially tumours, in which it functions as a pro‐oncogenic component. Moreover, in a variety of cancers, circ‐ZNF609 upregulation is often associated with poor prognosis. Functional experiments of cancer cells showed that circ‐ZNF609 could promote the proliferation, migration and invasion of tumour cells and inhibit cell apoptosis. In vascular thyroid dysfunction and CNV, the overexpression of circ‐ZNF609 can inhibit the formation of normal blood vessels and promote the formation of pathological blood vessels, which is correlated with the influence of circ‐ZNF609 on the function of relevant cells. In HSCR, overexpression of circ‐ZNF609 also inhibits cell proliferation and migration.

Salmena et al.^94^ proposed the theory of ceRNA for the first time that a miRNA can regulate multiple target genes, and the same target genes can also be regulated by different miRNAs. This hypothesis is on the basis of the fact that miRNAs can recognize their specific target sites (miRNA response elements, MRE) in different RNA molecules, contributing to target inhibition via miRNA and RNA‐induced silencing complex‐mediated degradation. Hence, miRNAs might mediate regulatory crosstalk between the diverse components of the transcriptome, comprising mRNAs, pseudogenes, lncRNAs and circRNAs. When mRNAs and circRNAs share the same MRE, they potentially compete for the same pool of miRNAs. Thus, when the expression level of circRNAs is increased, it could bind and titrate more miRNAs, remaining fewer miRNAs available for the corresponding mRNAs, so as to promoting the expression of downstream genes indirectly. Inversely, when circRNAs are downregulated because of a biological disturbance, the expression of target mRNAs might be decreased accordingly.

For ceRNA mechanistic studies, the circRNAs should be quantified using a circRNA‐specific method, such as RNase R and Actinomycin D assays. Then, Nuclear and cytoplasmic extraction assay and RNA FISH assay should be conducted to verify the subcellular localization of circRNA. As is known to all, circRNAs perform the function of ceRNA mainly in the cytoplasm. Besides, bioinformatics prediction, luciferase reporter assay, RNA immunoprecipitation and RNA pull‐down should be conducted to explore circRNA‐miRNA interactions.

However, the results presented in many original papers involved in this review always lack very important controls. Legnini et al.^8^ demonstrated that circ‐ZNF609 is related to heavy polysomes, and it could be translated into a protein in a splicing‐dependent and cap‐independent manner, while most of the studies did not explore the protein‐coding potential of circ‐ZNF609. Besides, FISH assay could more intuitively explore the localization of circ‐ZNF609 and miRNAs, while the results of nucleus‐cytoplasmic separation assay were not convincing. Moreover, most of the studies found the potential miRNAs which might bind to circ‐ZNF609 via bioinformatics prediction and verified the two RNA molecules interact to each other by luciferase reporter and RNA Immunoprecipitation assay. However, pull‐down assay should be performed to analyse the interacting miRNAs of circ‐ZNF609, which is more convincing.

In several studies, the increased expression of circ‐ZNF609 is accompanied by the decreased expression of miRNAs. The corresponding upregulation of downstream genes is difficult to be explained whether the sponge binding effect of circ‐ZNF609 or the reduced degradation effect of miRNAs. Furthermore, the circ‐ZNF609 mutant type should be constructed to be transfected with cell and explore its potential effects on cell function and downstream genes.

Recent studies have shown that circRNA is enriched in exosomes and plays an important role in intercellular communication.^95^ Exosomal circRNAs are stable in blood. Serum exosomal circRNA has been well documented to distinguish cancer patients from healthy controls and can be used as a biomarker for liquid biopsy.^96^ Shang et al.^97^ revealed that the cancer‐derived exosome circ‐PACRGL plays an oncogenic role in the progression of CRC. Another study reported exosomal circ‐SHKBP1 could promote GC progression and serve as a promising circulating biomarker for GC diagnosis.^98^ However, the role of circ‐ZNF609 in exosomes remains to be explored.

Taken together, these studies highlight the molecular mechanisms and biological functions of circ‐ZNF609 in various diseases. However, there are some limitations in these studies, such as a lack of rescue tests and limited sample size, and thus, the findings need to be further verified by independent cohort studies and multicentre studies. Besides, most of involved studies did not provide strong theoretical support to the sponge hypothesis and the experiments provided to support such models are based on inconclusive data. In addition, future studies should focus on its translational application in clinical diagnosis and prognosis assessments.

## CONFLICT OF INTEREST

The authors have declared that no competing interest exists.

## AUTHOR CONTRIBUTION

**Yiguan Qian:** Data curation (lead); Formal analysis (lead); Investigation (equal); Methodology (equal); Project administration (equal); Visualization (equal); Writing‐original draft (lead); Writing‐review & editing (equal). **Yang Li:** Data curation (lead); Formal analysis (lead); Investigation (equal); Methodology (equal); Project administration (equal); Resources (equal); Validation (equal); Writing‐original draft (equal); Writing‐review & editing (equal). **Rongfei Li:** Data curation (equal); Formal analysis (equal); Project administration (equal); Validation (equal). **Tianli Yang:** Data curation (supporting); Validation (equal); Visualization (supporting). **Ruipeng Jia:** Conceptualization (equal); Funding acquisition (supporting); Project administration (lead); Writing‐review & editing (equal). **Yu‐Zheng Ge:** Conceptualization (lead); Data curation (equal); Formal analysis (equal); Funding acquisition (lead); Investigation (equal); Methodology (equal); Project administration (lead); Resources (equal); Software (equal); Supervision (lead); Validation (equal); Visualization (equal); Writing‐original draft (equal); Writing‐review & editing (lead).

## Data Availability

Data sharing is not applicable to this review article as no new data were generated or analysed in this study.
